# Short-term diesel exhaust inhalation in a controlled human crossover study is associated with changes in DNA methylation of circulating mononuclear cells in asthmatics

**DOI:** 10.1186/s12989-014-0071-3

**Published:** 2014-12-09

**Authors:** Ruiwei Jiang, Meaghan J Jones, Francesco Sava, Michael S Kobor, Christopher Carlsten

**Affiliations:** Centre for Molecular Medicine and Therapeutics, University of British Columbia, 950 west 28th Avenue, Vancouver, V5Z4H4 Canada; Air Pollution Exposure Laboratory, Chan-Yeung Centre for Occupational and Environmental Lung Disease, Department of Medicine, Division of Respiratory Medicine, University of British Columbia, 2775 Laurel Street, Vancouver, British Columbia V5Z1L9 Canada; Human Early Learning Partnership, School of Population and Public Health, University of British Columbia, 2206 East Mall, Vancouver, British Columbia V6T1Z3 Canada

**Keywords:** Particulate matter, Air pollution, Epigenetics, Interspersed repetitive sequences, microRNAs

## Abstract

**Background:**

Changes in DNA methylation have been associated with traffic-related air pollution in observational studies, but the specific mechanisms and temporal dynamics therein have not been explored in a controlled study of asthmatics. In this study, we investigate short-term effects of diesel exhaust inhalation on DNA methylation levels at CpG sites across the genome in circulating blood in asthmatics.

**Methods:**

A double-blind crossover study of filtered air and diesel exhaust exposures was performed on sixteen non-smoking asthmatic subjects. Blood samples were collected pre-exposure, and then 6 and 30 hours post-exposure. Peripheral blood mononuclear cell DNA methylation was interrogated using the Illumina Infinium HumanMethylation450 Array. Exposure-related changes in DNA methylation were identified. In addition, CpG sites overlapping with Alu or LINE1 repetitive elements and candidate microRNA loci were also analyzed.

**Results:**

DNA methylation at 2827 CpG sites were affected by exposure to diesel exhaust but not filtered air; these sites enriched for genes involved in protein kinase and *NFkB* pathways. CpG sites with significant changes in response to diesel exhaust exposure primarily became less methylated, with a site residing within *GSTP1* being among the significant hits. Diesel exhaust-associated change was also found for CpG sites overlapping with Alu and LINE1 elements as well as for a site within *miR-21*.

**Conclusion:**

Short-term exposure to diesel exhaust resulted in DNA methylation changes at CpG sites residing in genes involved in inflammation and oxidative stress response, repetitive elements, and microRNA. This provides plausibility for the role of DNA methylation in pathways by which airborne particulate matter impacts gene expression and offers support for including DNA methylation analysis in future efforts to understand the interactions between environmental exposures and biological systems.

**Electronic supplementary material:**

The online version of this article (doi:10.1186/s12989-014-0071-3) contains supplementary material, which is available to authorized users.

## Background

Exposure to air pollutants is an increasing public health concern that has been associated with adverse health effects focused on cardiovascular and respiratory diseases [1–3]. Traffic-derived pollution appears particularly toxic, perhaps due to its abundance of fine particulate matter (PM) [4]. Ambient PM is a heterogeneous mix of components varying in concentration, and chemistry [5]. PM is associated with both chronic and acute effects on health [5], including increased risk of lung cancer [6–8], increased hospital admission rate for respiratory and cardiovascular diseases in both adults and children [3,5,9], and increased risk for mortality [10]. In urban environments, the major contributor to fine PM (diameter between 0.1 μm and 2.5 μm) is diesel exhaust (DE) [11]. Due to its small size, fine PM can deposit deep in the lung, and its soluble components such as transition metals may cross the lung epithelium into systemic circulation and interact with internal organs [2]. On the cellular level, exposure to DE prompts the generation of reactive oxygen species, leading to oxidative stress and damage to cellular structures [12–14]. On the molecular scale, DE has been found to change microRNA expression, increase production of allergic antibodies, up-regulate mRNA expression of pro-inflammatory mediators and antioxidant enzymes, as well as decrease methylation of repetitive genomic elements [15–19]. The hazardous effects of DE is also associated with asthma susceptibility and severity; for example, evidence links exposure to diesel exhaust with worse lung functions and increased airway resistance [20,21]. However, the precise mechanism by which pollution exacerbates asthma is not yet fully understood [5]. It has also been hypothesized that exposure to DE may be partly responsible for the increase in allergic diseases in industrialized nations [22].

One possible mechanism through which air pollution impacts transcriptional pathways may be exposure-related epigenetic modifications. Epigenetics refers to persistent changes in gene regulation that do not involve changes in DNA sequence [23]. Arguably the most studied and best-understood epigenetic modification is DNA methylation, the covalent addition of a methyl group to a cytosine primarily in the context of a cytosine-guanine dinucleotide (CpG). DNA methylation modulates gene expression, and can vary in response to external stimuli [23–26]. DNA methylation is also commonly observed at repetitive elements. LINE1 and Alu generally have higher methylation than the rest of the genome, and their methylation level is negatively correlated with mobility of retrotransposons [27]. Increased mobility of these retrotransposons, especially at cancer-related genomic loci, is associated with mutation and higher tumorigenesis rates [27]. Many recent studies have shown that ambient particulate matter change methylation of LINE1 and Alu repetitive elements as well as that of pro-inflammatory and tumor suppressor genes[28–31]. Thus methylation at repeat elements is responsive to environmental influences; this could affect chromosomal arrangements and gene expression, relating environment to disease.

These epigenetic responses to environmental exposures are not limited to repetitive elements and particulate matter [23,25,32]. DNA methylation is an important mechanism through which the environment in general interacts with the genome, likely affecting phenotypic outcomes. Environmental effects with known DNA methylation associations include socio-economic status, pollution, stress, and personal habits such as diet and smoking [16,23,33]. Although the mechanisms behind these associations are still unknown, it is undeniable that there is a complex dynamic at play among environment, genes and epigenetics. As such, epigenetics presents an intriguing target for understanding the mechanisms behind the adverse effects of DE.

Despite the numerous existing investigations regarding ambient particulate matter and DNA methylation, no controlled investigation of particulate matter on DNA methylation at sites distributed across the genome, an important starting point for unbiased mechanistic inquiry, has been reported. We were interested in understanding the systemic impact of air pollution from the perspective of DNA methylation, and thus focused on peripheral blood mononuclear cells (PBMCs). We hypothesized that short-term exposure to DE would lead to changes in DNA methylation status of PBMCs at CpG sites across the genome in asthmatic individuals, especially in genes relevant to the etiology of allergic diseases. Furthermore, we speculated that we would also observe changes in methylation of LINE1 and Alu repetitive elements, given that repetitive elements have been shown to be sensitive epigenetic indicators of environmental exposure [16,30]. Lastly, we investigated whether any methylation changes were linked to DE induced changes in microRNA expression that we have previously demonstrated in the same individuals tested here [19].

## Results

### Diesel exhaust was associated with changes in DNA methylation

**Figure 1:**
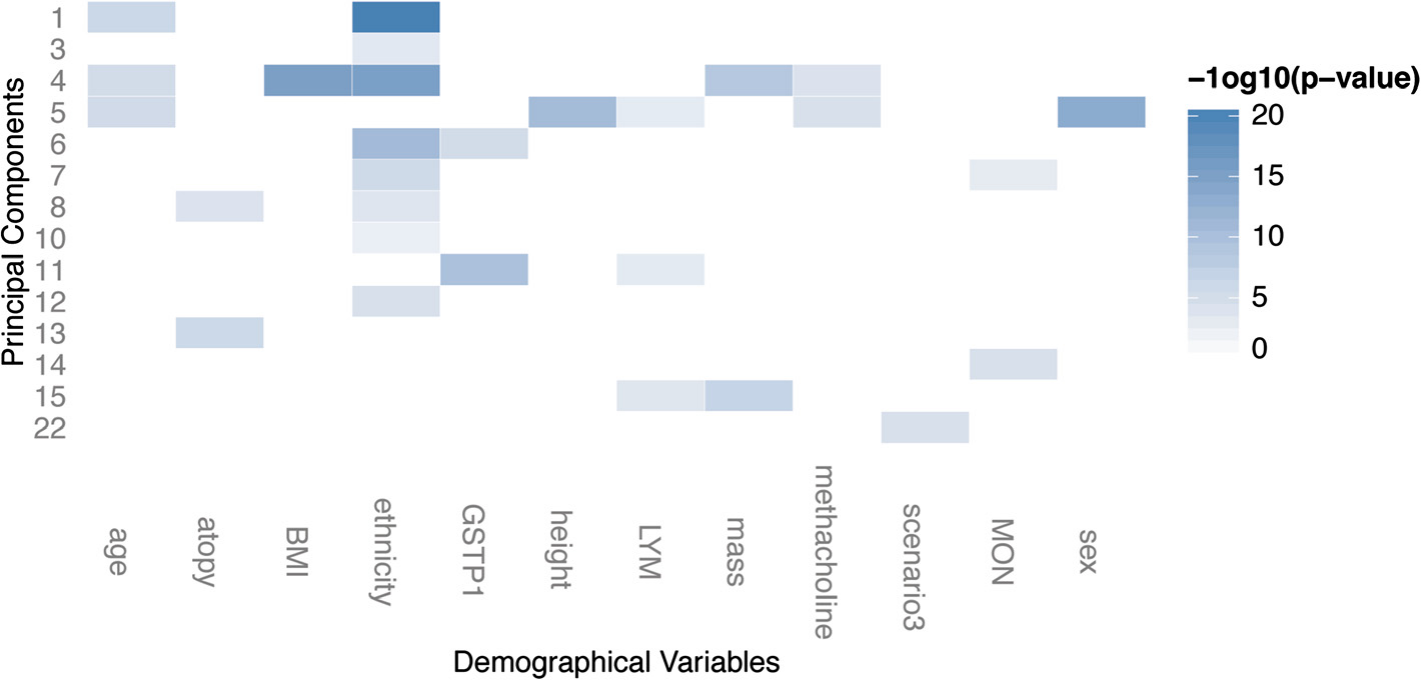
**Principal components were associated with demographics and biological variables.** For principal components 1-22, only those with associations were included. Colors correspond to p-values where darker colors indicate higher association, and lighter colors indicate lower association. Abbreviations are as follows: basophils (BAS), lymphocytes (LYM), monocytes (MON).

The demographic characteristics of the sixteen subjects with physician-diagnosed asthma and/or baseline methacholine PC_20_ below 8 mg/mL are in Table 1. Using PCA, a dimensionality reduction technique, we decomposed the data to a set of 95 principal components (PCs), each of which explained a dominant and independent pattern of variation across the samples; we then assessed the relative contribution of each principal component to the overall variance (see Additional file 1: Figure S1 in online data supplement). PCA facilitated linear decomposition of the data, allowing identification of data dimension that significantly captured the association between DNA methylation change and exposure to DE. By focusing our analysis on the CpG sites most representative of that dimension, we were able to analyze only CpG sites with higher chance of being DE-associated. Surveying the pattern of methylation within the data, we found that demographic variables were associated with a number of PCs, and many of the associations were driven by correlations inherent among these variables (see Additional file 2: Figure S2 in online data supplement; significant associations were captured after multiple testing correction by the q-value method with FDR = 10%). In particular, age was a major factor driving data variability in PCs 1, 4, and 5, and BMI was strongly associated with PC4 (Figure 1). Since PBMCs are heterogeneous, we considered the contribution of blood cell composition to methylation change. To achieve this, we tested for association between PCs and white blood cell counts. We did not find association between PCs and any granulocyte cell types, as expected since they are removed during PBMC isolation. However, monocyte and lymphocyte counts were positively associated with PCs 7 and 14, and PCs 5, 11, 15, respectively. This indicated that inter-individual difference in blood cell composition was also a source of variance in the dataset. To account for blood composition difference and avoid confounding between diesel related methylation change and change resulting from blood cell population shifts, we identified CpG sites that were significantly associated with changes in blood count. First we calculated the probe-wise loading values of PCs 5, 7, 11, 14 and 15; for CpG site *i* with loading value *m* for PC *n*, the larger the magnitude of *m*, the closer the methylation pattern of CpG *i* approximates PC *n*. Using cutoffs of +/-3SD, we found 19250 sites that were potentially associated with blood cell counts. By regressing DNA methylation measurements at these sites against lymphocyte and monocyte cell counts, we identified 11378 sites that were significantly associated with blood cell counts (multiple testing correction by the q-value method; FDR =10%). All hits reported to be associated with DE in this study have been filtered to exclude these sites associated with blood cell composition.

**Table 1:** **Subject demographics used with principal component scores to determine main drivers of variation in data**

**Variable**	**Measurements**
Gender: Male	7 [44%]^†^
Age, years	28.7 ± 6.7 [19.2-46.5]
Ethnicity	
Asian	2 [13%]^†^
Caucasian	12 [75%]^†^
Middle Eastern	1 [6%]^†^
South Asian	1 [6%]^†^
Height, cm	170.0 ± 12.2 [151.0-185.5]
Mass, kg	71.9 ± 15.5 [53.0-104.6]
BMI, kg/m^2^	24.8 ± 3.9 [19.8-34.7]
FEV1, % predicted	90.3 ± 14.1 [66.0-120.0]
Atopy: atopic	9 [56%]
Asthma diagnosis: yes	12 [75%]
Methacholine-responsive: yes	15 [94%]
GSTP1: G allele	11 [69%]
Lymphocytes, K/μL	1.9 ± 0.5 [1.2-2.6]
Monocytes, K/μL	0.43 ± 0.13 [0.20-0.70]
Basophils, K/μL	0.03 ± 0.05 [0.00-0.10]

Except where indicated, data are presented in terms of mean ± standard deviation with range or percentage of total in brackets.

^†^Count [percentage].

A lingering concern with DNA methylation time-series data is that there would an influence of time, stochastic or biological, that affects measurements. We did not find association of time (0 hr to 30 hr) with any PC, indicating that DNA methylation did not vary as a result of measurements taken across the three time points.

**Figure 2:**
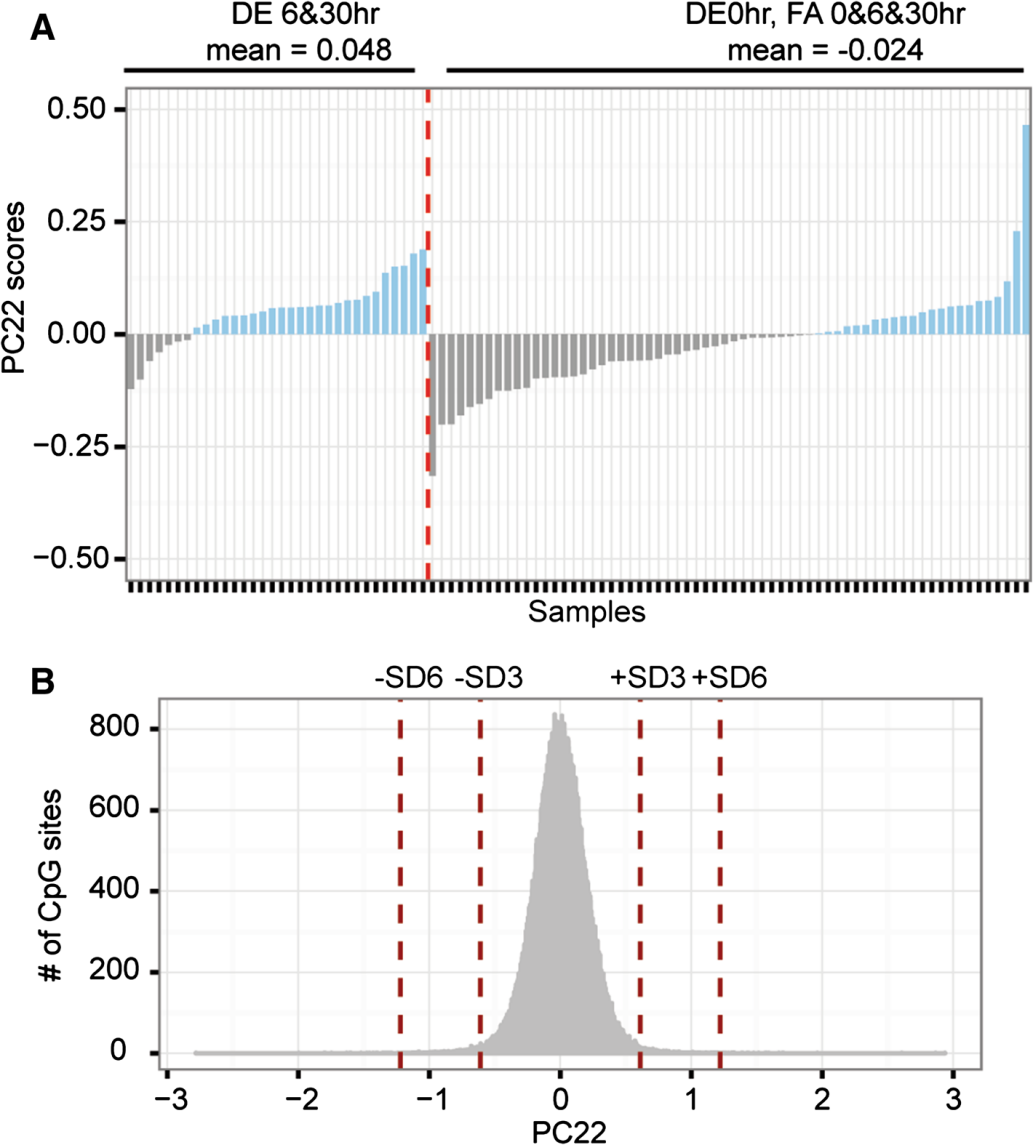
**Diesel exhaust-associated exposure patterns were captured in principal component 22. A)** PC22 was associated with model-3 (DE6&30 hr vs. non-DE6&30 hr). Samples were sorted according to DE6&30 hr samples versus non-DE6&30 hr baseline samples and their PC22 associated principal component scores were displayed. There was a visible pattern indicative of DE-exposure with the DE6&30 hr samples showing mostly positive scores, and the baseline samples showing mostly negative scores. **B)** For each probe in the dataset, its loading value in association with PC22 was calculated. The +/-3SD and +/-6SD positions are marked by vertical dashed lines. And the probes harboring the largest contribution to diesel exposure methylation pattern were selected for using the +/-3SD cut off, resulting in 2827 hits.

Having controlled for potentially confounding variables, we next sought to identify CpG sites associated with DE exposure. We assessed three different scenarios of DE-induced changes assuming baseline measurements at FA-0 hr, FA-6 hr, FA-30 hr, and DE-0 hr (serving as pre-exposure baseline), and tested for association between these scenarios and principle component scores using Wilcoxon’s ranked sum test. Scenario-1 hypothesized that methylation changes are detectable 6 hr post DE-exposure, but diminish at 30 hr post-exposure (thus, it compared DE6hr against non-DE6hr). Scenario-2 hypothesized that changes are detectable 30 hr post-exposure (thus, it compared DE30hr against non-DE30hr). Scenario-3 hypothesized that changes are detectable at 6 hr and persist at 30 hr post-exposure (thus, it compared DE6hr&30 hr against non-DE6hr&30 hr). Scenario-3 was significantly correlated with PC22, which accounted for 0.6% of the total variance. Importantly, PC22 was not associated with any experimental confounds such as demographic factors and blood cell counts. To further examine the pattern of variation underlying PC22, we derived the PC22 scores, which could be used to interpret the correlation between that PC and variable Scenario-3. By ordering the PC22 scores into DE6hr&30 hr versus non-DE6hr&30 hr, we observed that DE6hr&30 hr samples exhibited mostly positive scores with a mean of 0.048, whereas baseline samples exhibited mostly negative scores with a mean of -0.024. This observation was statistically substantiated by a two-sample t-test (p-value = 1.6e-4) (Figure 2A). Next, we identified DE-associated CpG sites by calculating the probe-wise loading values of PC22 (Figure 2B). Using cutoffs of +/-3SD, we found 2827 such sites. Functional analysis of these probes using DAVID revealed enrichment of genes involved in regulation of protein kinase and *NFkB* pathways (enrichment score of 3.01; Additional file 3: Table S1) [34].

### DE-associated changes were found in genes relevant to allergic disease

**Figure 3:**
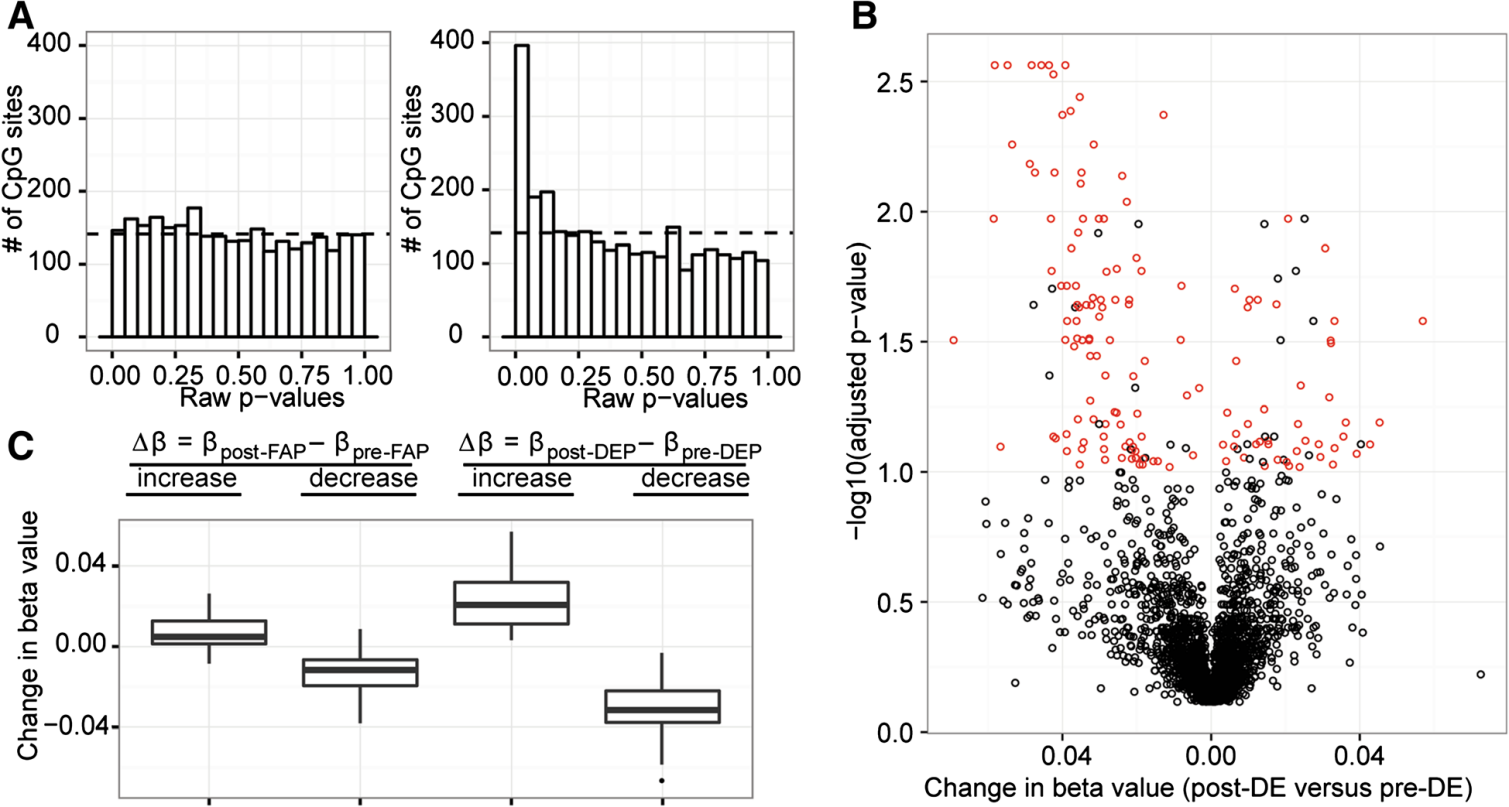
**Gene-specific methylation changes were found at CpG sites across the genome. A)** Unadjusted LME model p-value distributions for the effect of pre (0 hr) versus post (6-30 hr) exposure for FA, and that for DE. The uniform distribution expected by chance is indicated with horizontal dashed lines. Lack of positive skewing in the FA distribution indicated no association between FA exposure and differential methylation, whereas positive skewing in the DE plot indicated heavy association of DE exposure with differential methylation. **B)** Volcano plot of 2827 PCA hits. Negative log10 of fdr 10% adjusted p-values from LME modeling of the PCA hits were visualized against their corresponding ∆β values. Probes that were significant with a high confidence fdr of 10% were indicated in red. **C)** Distribution of ∆β obtained from subtracting pre-FA beta values from post-FA beta values, and from subtracting pre-DE beta values from post-DE beta values for the PCA identified probes that were significantly altered by DE exposure. ∆β from DE samples were much larger in magnitude than that from FA samples.

The deviation from random of the raw p-value distributions showed that the post-DE (6 hr&30 hr) versus pre-DE (0 hr) comparison demonstrated high association with DNA methylation change, suggesting that they were more correlated than that expected by chance, while the same association was not found for post-FA versus pre-FA (Figure 3A). Consistent with the pronounced skewing of the p-value distributions, 170 differentially methylated positions (DMPs) were significant for DE exposure (after multiple testing correction using high confidence FDR of 10%), but not FA exposure. Out of these 170 sites, 25 were previously identified to be significantly associated with blood cell counts; these sites were removed from proceeding analysis, leaving 145 DMPs that demonstrated significant change in methylation as a result of exposure to DE. Specifically, 102 out of the 145 DMPs decreased in methylation (Additional file 3: Table S2a), while 43 sites increased in methylation (Figure 3B and C, Additional file 3: Table S2b). We calculated the average beta value change (Δβ) between post-exposure and pre-exposure for the DMPs, and we found 1 probe showing increase and 6 probes showing decrease in DNA methylation of greater than 5%. Interestingly, 1 out of 8 *GSTP1* sites (cg09038676) were among the significantly correlated loci. For *GSTP1*, we genotyped subjects for the A- > G substitution at position 313 of the *GSTP1* gene, and we used this information to stratify the samples in an attempt to determine whether the difference in magnitude of DNA methylation change was related to genotype. As expected, at baseline under the FA condition, DNA methylation levels at cg09038678 for subjects with the G allele were significantly different from that of subjects with the A allele (two-sample t-test p-value = 0.00024). Furthermore, individuals with the A allele showed significant change pre- and post- DE exposure, but not individuals with the G allele (see Additional file 4: Figure S3 in online data supplement).

Lastly, we tested whether DE-related effects carried over to the subsequent FA exposure. Hypothetically, if carryover effects were present then probes found to have increased methylation would show higher mean FA methylation in subjects exposed to diesel first (vice versa for probes found to have decreased methylation). We compared FA mean methylation values in participants who were exposed to DE before FA by first separating the 145 DMPs into ones with decreased and increased methylation in response to DE, then separating the groups further according to exposure order (see Additional file 5: Figure S4 in online data supplement). Neither visual inspection of the graphs nor Welch’s two-sample t-test did support any carryover effect (p-value > 0.05), consistent with our previous assessment of effective binding [35].

### Alu and LINE1 CpG sites exhibited methylation change post DE exposure

**Figure 4:**
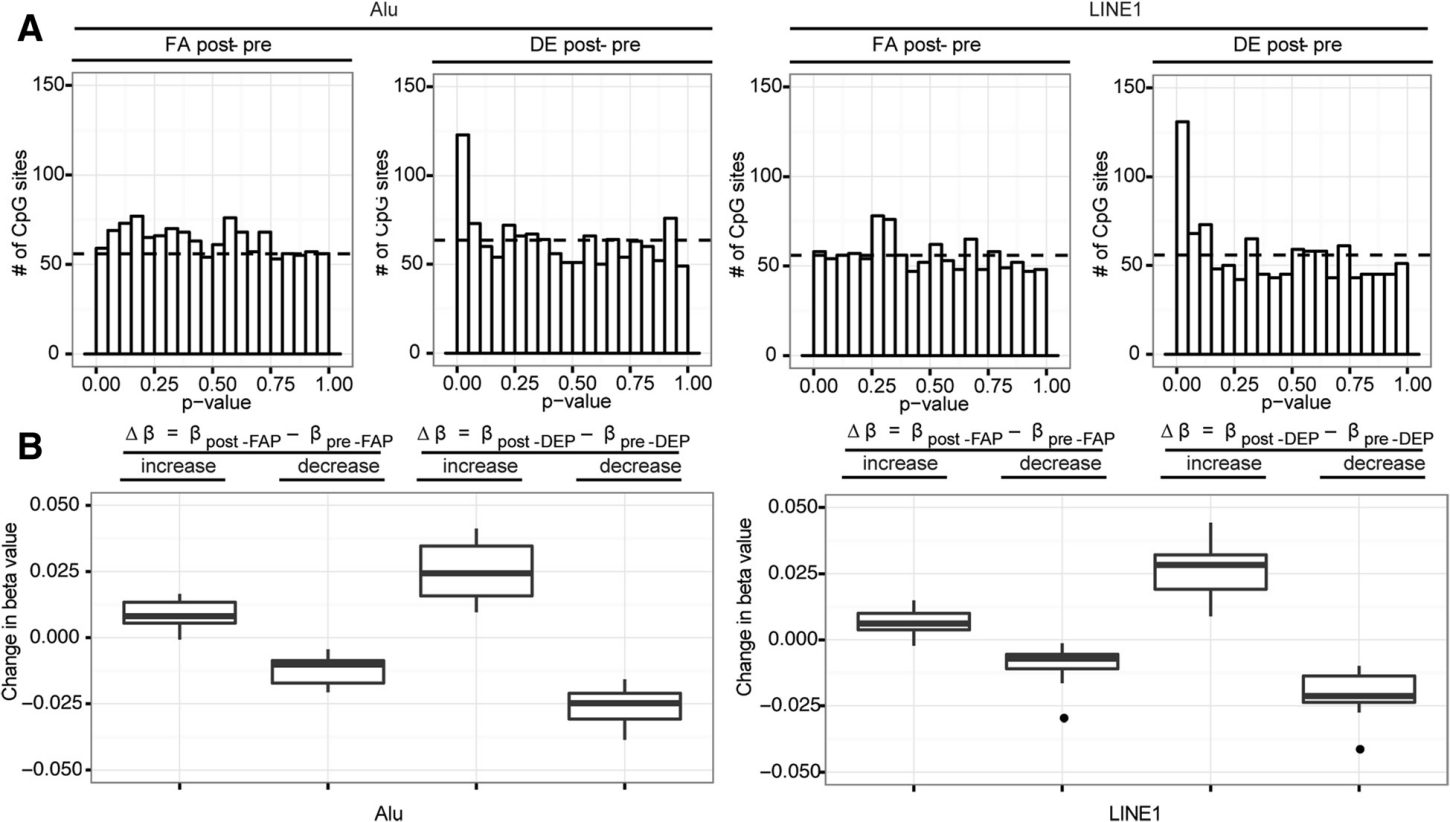
**DNA methylation of CpG sites overlapping with Alu and LINE1 repetitive elements was associated with diesel exhaust. A)** Distributions of unadjusted p-values from LME modeling applied to CpG sites overlapping with Alu and LINE1 elements. The uniform distribution expected by chance is indicated with horizontal dashed lines. Positive skewing of DE p-value distributions indicated association with differential methylation, whereas lack of skewing of FA p-value distributions indicated absence of association. **B)** Distributions of ∆β obtained from subtracting pre-FA beta values from post-FA beta values, and from subtracting pre-DE beta values from post- DE beta values for LINE1 and Alu probes that were significantly altered in response to DE exposure. ∆β from DE samples were much larger in magnitude than that from FA samples.

To investigate the impact of DE-exposure on the methylation of Alu and LINE1, we identified *in silico* all probe positions on the array that shared a larger than 15 base-pairs overlap with Alu or LINE1 repetitive elements in the genome. Applying LME modeling to these CpG sites, both Alu and LINE1 elements exhibited changes in DNA methylation as a result of DE exposure, and no changes as a result of FA exposure (Figure 4). 25/1271 (2%) and 31/1118 (3%) DMPs (CpG sites significantly associated with blood cell counts were not considered DMPs) were identified for Alu and LINE1, respectively (Additional file 3: Table S2c, Table S2d). Out of the 25 Alu DMPs, 12 increased in methylation while 13 decreased in methylation after DE exposure; similarly for the 31 LINE1 DMPs, 13 increased while 18 decreased in DNA methylation after exposure. The significant loci found for Alu and LINE1 elements did not overlap with the 145 PCA DMPs, since none of their PCA loading values made the ±3 SD cutoff.

### MiR21 showed decrease in methylation upon DE exposure

**Figure 5:**
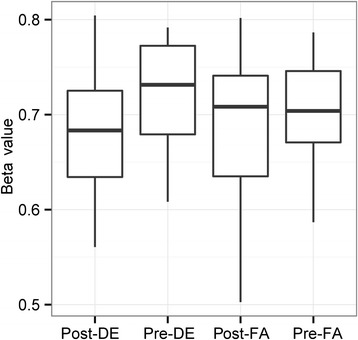
**A CpG site residing in the** ***miR-21*** **genomic locus changed in DNA methylation in response to DE.** Post and pre methylation beta values of CpG site cg07181702 (residing within *miR-21*) found to have significantly decrease in methylation by 3.9% in response to DE-, but not FA-, exposure.

Recently we identified changes in expression in peripheral blood of four microRNAs (*miR-21*, *miR-30e*, *miR-215* and *miR-144*) in response to DE exposure in the same subjects that were involved in this study [19]. To determine whether differential methylation could be a mediator for the expression of these four microRNAs, we localized a total of 7 probes on the array that overlapped with the genomic positions of these microRNAs. A probe residing within *miR-21* (cg07181702), which demonstrated increase in expression in response to DE, showed significant decrease in methylation in this study (and absence of significant change with FA): cg07181702 decreased in methylation by 3.9% in response to DE exposure (significant with 10% FDR) (Figure 5)[19].

## Discussion

Inhaled air pollution including emissions from diesel engines have been associated with a host of cardiovascular and respiratory diseases, imparting a significant strain on public health [3,9]. Data presented here on DNA methylation changes in response to short-term exposure to DE demonstrated a potential epigenetic mechanism for biological responses to DE exposure. PCA enabled detection of DE signals from subject-level noise generated by demographic variables such as age, ethnicity, and BMI, and focused the investigation onto a smaller list of pertinent CpG sites. Furthermore, the DE-associated methylation changes in LINE1 and Alu CpG sites corroborated with previous research demonstrating sensitivity of repetitive elements to environmental exposure [16,29]. Lastly, methylation change was also observed for *miR-21*, a microRNA associated with oxidative stress and allergic inflammation. Collectively, these results were consistent with our hypothesis that epigenetics servers as a potential avenue through which exposure to air pollution impacts biological systems.

Air pollution has long been linked to diseases including asthma and cardiovascular issues [1,3,32,33]. What is still lacking in our understanding is defining the biological pathways that may be either a mediator or a consequence of the association. DE is a major source of fine particles that have been shown to impact gene expression, and change DNA methylation at inflammation associated sites [16,28,29,31]. Our results support previous studies that have found an association between PM exposure and decrease in DNA methylation of target promoter, candidate genes and repetitive elements [16,29,30] We suggest that this could be explained through the effects of DE on the dynamics between DNA and DNA methyltransferases. First, ROS-induced oxidative damage to methyl-CpG binding protein recognition sequences could have inhibited the capacity for DNA methyltransferase to bind and methylate DNA [36]. Secondly, decrease in saturation of the DNA methyltransferase enzymes could potentially come into play, since studies have shown a dependent relationship between exposure to particles and decrease in mRNA transcripts of the DNA methyltransferases *Dnmt1*, *Dnmt3a*, *Dnmt3b* in mouse macrophages [37]. Decreased mRNA transcripts could lower the cellular concentration of methyltransferases and as a result decease the methylation level of CpG sites. Both of these scenarios could be part of the mechanism leading to loss of methylation that partially explains why the majority of the gene-specific sites found here showed decrease in methylation.

DAVID functional enrichment analysis of the 2827 DE-associated probes identified by PCA revealed enrichment of *NFkB*-related functions. *NFkB* is a redox-related transcription factor that activates a pro-inflammatory response to ROS induced oxidative stress [38–41]. *NFkB* has been shown to activate in response to DE exposure, increasing the downstream expression of inflammatory cytokines [42]. Besides *NFkB*, we also discovered enrichment of the MAPK protein kinase pathway. Previous studies have shown that MAP kinase is a stress-activated kinase pathway that can be induced by DE exposure [12,43]. Given the proposed role of DNA methylation as a mediator between environment and gene expression, the observations made here further demonstrated that a tight circuitry exists between transcriptional pathways known to be involved in response to DE and DNA methylation changes elicited by DE exposure.

Results from PCA followed by regression modeling in this study revealed that in agreement with our hypothesis, DE was associated with changes in DNA methylation at genes that are known to be associated with inflammation and oxidative stress, most notably *GSTP1* [44,45]. Studies investigating the effects of *GSTP1* gene polymorphism found that subjects homozygous for the *GSTP1* G allele have lower functional levels of the enzyme, and thus are at increased risk for oxidative stress, lung cancer, and asthma [44,46,47]. Since the *GSTP1* associated probe (cg09038676) was located in the body of the *GSTP1* gene, then its increase in DNA methylation would likely to be associated with increase in *GSTP1* transcription, resulting in higher levels of the enzyme to combat the effects of oxidative stress. Interestingly, this effect was most profound for subjects with at least one A allele, suggesting that these individuals are less susceptible to the negative effects of DE exposure than individuals homozygous for the G allele, consistent with the literature [48]. Non-asthmatic controls were not included in this study, thus we cannot conclude that the changes observed are specific to asthmatics, but our finding bolsters mechanistic plausibility nonetheless. Furthermore, the significant baseline (FA) methylation difference between subject with and without the *GSTP1* G allele substitution that cg09038676 could have been a methylation associated SNP; however, due to the fact that only 5 of 16 subjects had an A allele, we were not able to quantitatively assess this possibility.

Besides *GSTP1*, we also found decreased methylation in a probe residing within the body of *HDAC9* (cg24458314), a class IIa histone deacetylase [49]. A study involving regulatory T (Treg) cells in mice showed that absence of *HDAC9* enhances the suppressive ability of Treg cells, resulting in decreased immune responsiveness and inflammation [49]. Changes in DNA methylation of HDAC9 such as we observed could impact downstream gene expression that then modifies the allergic airway inflammation in response to DE exposure. Considering that Tregs constitute only a small fraction of PBMCs, we speculate this small change might indicate a profound effect on the Treg population; however, a more concrete conclusion could not be reached without independent examination of Treg cell-specific methylation.

Lastly, we discovered that cg05094429, which resides in the promoter region of *CCR6* gene, decreased in methylation after exposure to DE. *CCR6* is expressed by both Treg and Th17 cells, and it is a key regulator of the migration of these cells to sites of inflammation [50]. Lack of this protein in Th17 cells hampers the recruitment of both Th17 and Tregs [50]. Thus it is possible that decreased methylation of *CCR6* in the promoter region could have resulted in increased expression of *CCR6*, eventually leading to increased presence of Th17 and Treg cells responding to DE induced inflammation. It should be noted an effect of ambient particulate matter on DNA methylation patterns has been previously documented in numerous studies [16,28–31]. Therefore, despite the fact that most changes found here were small in magnitude, the overall findings reported here demonstrated an effect of DE that is concordant with existing observations and is impactful on the genomic scale.

Repetitive elements comprise of half of the genome and, under normal conditions, harbor higher DNA methylation in comparison to the rest of the genome [51]. Repetitive elements are activated during cellular stress, which in this case could be elicited by exposure to DE [52]. In accordance with past research on effects of particulate matter, we found both increase and decrease in Alu and LINE1 methylation after DE exposures [37]. In some cases, demethylation of Alu and LINE1 increases genomic instability, which could mean that DE exposure predisposes some cells to genomic rearrangements [53–55]. Furthermore, repeat elements also impact adjacent sequences; they can propagate the spread of DNA methylation to nearby sequences or serve as insulators [56,57]. A recent study has shown that sequences close to a 3′ repeat element demonstrated better methylation stability [58] Therefore, it is conceivable that disruption of repetitive element DNA methylation due to DE exposure could be associated with genomic rearrangement as well as affecting methylation stability of nearby sequences causing changes in gene expression.

In this study we also examined the effects of DE on methylation of microRNAs, which are a class of small noncoding RNAs with functions in post-translational regulation of expressed genes that are important mediators of cellular processes [59]. Pollutants can cause microRNA dysregulation, which could lead to lung diseases and inflammation [19]. In particular, *miR-21* is involved in oxidative stress and allergic inflammation, and has been shown to be up-regulated in asthma [19,60–62]. Our results showing that DNA methylation was also altered after DE build upon a previous study on the same individuals that reported changes in *miR-21* expression upon DE exposure [19], though this observation was associative and we cannot conclude that the changes in methylation are directly responsible for the changes in expression.

Lastly we were able to demonstrate that DE-associated changes lasted at least 30 hr post-exposure. This observation was consistent with reports showing that methylation changes elicited by environmental exposure could persist for days in the absence of persistent triggers, though the precise dynamics of this in the context of air pollution requires further study. These results should be interpreted in light of the limited sample size, lack of another dataset sufficiently similar to attempt validation, and the systemic nature of the tissue investigated. Nevertheless, this investigation presents a novel approach to analyzing the association between PM and genome-wide DNA methylation, and although we did not observe residual effects in the subjects after 2 weeks, long-term or repeated exposures to DE may lead to accumulative effects. Future studies could build upon the approach presented here to investigate similar events in airway tissues.

## Materials and methods

### Study demographics

Sixteen participants were recruited at the Air Pollution Exposure Laboratory (APEL) in Vancouver, British Columbia, Canada. Written consent was obtained from all subjects, and the protocol was approved by the institutional review board for human studies at the University of British Columbia (H08-02288). Participants were 19-to 35-year-old nonsmokers who had had physician-diagnosed asthma for at least 1 year and/ or a methacholine challenge with ≤ 8 mg/mL in terms of the PC20, a provocative concentration of methacholine that induces a 20% fall in forced expiratory volume in 1 sec (FEV1). All participants were stable in terms of asthma symptoms [assessed by the asthma control questionnaire] and were free of respiratory infections for 4 weeks prior to and during the study period [63]. The participants were free from current use of inhaled corticosteroids, regular use of bronchodilator, and use of vitamin A, C, or E supplements. Throughout the study, participants were asked to withhold long-acting β2-agonists 48 hr prior to spirometry, short-acting β2-agonists 8 hr prior to spirometry, and caffeine 4 hr prior to methacholine challenges. The participants maintained a stable diet, including intake of cruciferous vegetables, over the course of the study; thus, in the context of the crossover design, diet was not considered confounding.

### Exposure design and procedures

Details of the exposure were previously documented [64]. This study followed a double-blind, crossover design in which each subject was subjected to either filtered air (FA) or diesel exhaust (nomimally, 300 μg/m^3^ PM_2.5_) for 2 hours on two separate occasions at least two weeks apart. The DE concentration was, on average, 301 μg PM2.5/m3 (SD of 16 μg/m3). The exposure booth temperature was maintained at 20 degrees Celcius and the relative humidity at 40 percent for all exposures. The sequence of filtered air or diesel exhaust exposure was randomized and counterbalanced, and exposures took place within the same season for a given subject. During exposures, subjects alternated between light exercise (15 min) and rest (45 min) on a stationary bicycle. The wattage of the stationary bike was calibrated in each individual to a minute ventilation of 15 L · min-1 · m^2^ body surface area.

Blood was collected in EDTA and Vacutainer® CPT™ tubes (BD Biosciences, Franklin Lakes, NJ) immediately before exposure as well as 6 and 30 hours after exposure onset and processed within 4 hours. CBCs were performed on whole blood prior to PBMC isolation. PBMC separation was performed following the protocol suggested by the manufacturer, after which PBMCs were stored at -80°C for analysis. Each subject was assessed for *GSTP1* rs1695 SNP genotype by PCR-Restriction Fragment Length Polymorphism using DNA isolated from whole blood.

### DNA methylation analysis

All procedures conducted using commercially available kits were done so following the manufacturers’ protocol. Two μg of genomic DNA per sample was extracted from PBMCs using the DNeasy Blood & Tissue Kit (Qiagen, Valencia, CA, USA). One μg of the purified DNA was then bisulfite-converted using the EZ-DNA methylation kit (Zymo Research, Orange, CA, USA), which changed epigenetic data into sequence-based data by the selective conversion of unmethylated cytosines to thymidines. Bisulfite-converted DNA was assessed for concentration and quality using the NanoDrop, and 4 μL of the conversion product was used for genome-wide DNA methylation evaluation at over 485,000 CpG sites using the Illumina Infinium HumanMethylation450 BeadChip array in house as described before [65].

### Array quality control and normalization

A total of 16 subjects were included in this study, distributed randomly across two chips. There were 6 samples for each subject (3 DE and 3 FA), making a total of 96 samples. Illumina GenomeStudio software was used to interpret array output, and signalA, signal, and probe intensity were exported into R for further processing and analysis. For each sample, probes with one or more samples that had undesirable detection p-values (p-value > 0.01) or with one or more missing measurements were removed (58482 sites). Then probes residing on the X or Y chromosome were also removed to control for gender-derived differences in the array (11648 sites). Lastly, probes that cross-hybridize to somatic sites or to sites on the X or Y chromosomes, as well as probes that possibly reside at SNP sites (as defined by Illumina annotation or independent re-annotation) were removed (52042 sites), regardless of their allele frequency in the population, leaving 363340 probes and 96 samples from 16 subjects for analysis [66]. Such stringent filtration method was applied to ensure that methylation measurements of the CpG sites investigated in this study were most representative of the larger population.

Chip to chip color bias correction was performed in R using the built-in color-correction and background subtraction, and quantile normalization functions of the lumi package with default settings. Finally, we applied peak-based correction method which has been reported to improve data accuracy and reproducibility. Furthermore, peak based correction improves detection rates of differentially methylation at CpG sites that would otherwise have been missed [67–69]. Two distinct values of DNA methylation were calculated, beta-values and M-values. Beta-value has a range of 0 to 1 and approximately represents percent methylation. M-values are log transformation of beta-values, and are more statistically robust. Thus all statistical analyses were performed using M values while visualization and discussion were presented using beta-values. The data obtained in this study has been deposited in the Gene Expression Omnibus repository under the accession number GSE56553.

### Identification of Alu, LINE1, and microRNA CpG sites

To identify probes overlapping with Alu and LINE1 repetitive elements, the repeat element track was downloaded from the University of California Santa Cruz (UCSC) Genome Browser (http://genome.ucsc.edu), and probes having at least 15 base pairs (bp) overlap with Alu or LINE1 elements in the genome were identified; given that the average Illumina probe is 50 bp in length, this coverage would ensure that at least 30% of the probes resides within the repetitive elements. Furthermore, after testing multiple cutoffs, we found that a cutoff of 15 bp gave the desirable number of Alu or LINE1 associated probes to represent coverage of Alu and LINE1 elements across the genome. A total of 7 probes overlapping with microRNAs *miR-21*, *miR-30e*, *miR-215* and *miR-144* were also identified and used in subsequent analysis.

### Principal component analysis

Mean normalization was conducted across samples, and the prcomp function from the R stats package was used to perform principal component analysis on the dataset. The 0^th^ principal component (PC) accounted for the difference in global probe intensity from one probe to the next and thus was not considered. To reduce the number of random variables under consideration, tests for association between experimental variable and PCs were carried out. Specifically ANOVA was used for nominal variables (time, subject, ethnicity, chip number, location on chip), spearman correlation was used for continuous variables (Height in cm, mass in kg, BMI, FEV1, age), and Wilcoxon-ranked sum test was used for dichotomous variables (FA vs. DE, DE hr6 vs. non-DEhr6, DEhr30 vs. non-DEhr30, DEhr6&30 vs. non-DEh6&30, sex, atopy, asthma, methylation, *GSTP1* genotype). To control for type II error, Storey’s qvalue method was used with false discovery cutoff of 0.1 (high confidence) [70].

### DAVID functional enrichment analysis

UCSC refgene accession-IDs corresponding to the 363340 CpG sites involved in this analysis was used as background for DAVID GO analysis (http://david.abcc.ncifcrf.gov/) [34]. The 2827 CpG sites were tested for enrichment of GO subcategories biological process and cellular component. Functional enrichment scores of larger than 1.3 were considered significant.

### Linear regression modeling:

The following linear regression model was applied to the 11378 lymphocyte and monocyte-count associated CpG sites identified by principal component analysis.

$$ {Y}_{ij}={\upbeta}_0+{\upbeta}_1(Lymphocyte) + {\upbeta}_2(Monocyte)+{\upvarepsilon}_{ij} $$

This model was used to identify sites that were significantly associated with blood cell counts. *Y*_*ij*_ represented the measured methylation value of individual *i* at CpG site *j. β*_*0*_ represented the overall intercept, *β*_*1*_ represented the effect of lymphocyte count on methylation value, *β*_*2*_ represented the effect of monocyte count, and *ε*_*ij*_ represented the error term.

### Linear mixed effects modeling

The following linear mixed effects model was applied to principal component analysis identified DE-associated CpG sites, as well as sites overlapping with Alu, LINE1, and microRNA sites of interest using the lme function from the nlme package [71]. Since PC22 was associated with only diesel exposure, and it did not show confounding with any covariates, only exposure associated variables needed to be included in this model.

$$ {Y}_{ijkl}={\upbeta}_0+{\upmu}_i+{\upbeta}_1(Exposure) + {\upbeta}_2(Time)+{\upbeta}_3\left( Exposure\times Time\right)+{\upvarepsilon}_{ijkl} $$

The model was used to test for change in methylation due to diesel exhaust exposure, assuming random intercept according to subject. *Y*_*ijkl*_ represented the measured methylation value of individual *i*, at CpG site *j*, exposure *k* and time l. *β*_*0*_ represented the overall intercept, *μ*_*i*_ represented the random intercept of individual *i*, *β*_*1*_ represented the main effects of exposure, *β*_*2*_ represented the main effects of time (pre-exposure 0 hr vs. post exposure 6&30 hr), and *β*_*3*_ represented the interaction effect between exposure and time, and lastly, *ε*_*ijkl*_ represented the error term. This model was used to identify probes that changed in methylation as a result of exposure to DE but not FA.

## Additional files

Additional file 1: Figure S1. Percent variance within the dataset accounted for by the first 22 principal components (0^th^ principal component was disregarded since it is focused on probe offsets). Additional file 2: Figure S2. Heatmap showing correlation among demographic variables and differential cell counts (please see Table 1 for units). Red indicates negative Pearson’s correlation coefficient of -0.5. Green indicates positive Pearson’s correlation coefficient of 1.0. Abbreviations are as follows: basophils (BAS), lymphocytes (LYM), monocytes (MON). Additional file 3: Table S1. Top 4 DAVID functional annotation clusters. Enrichment of cellular component, biological process, and molecular function in the 2827 PCA filtered probes was calculated using all probes involved in this analysis as the background. **Table S2.** CpG sites found to have significant decrease in methylation as a result of DE exposure through LME modeling. For each probe, ∆β(FA6&30hr-FA0hr) represents the change in beta value from pre-FA exposure at 0hr and post-FA exposure at 6 and 30hr. ∆β(DE6&30hr-DE0hr) represents the change in beta value from pre-DE exposure to post-DE exposure. Closest TSS gene name indicates the gene name of the closest transcription start site. UCSC RefGene name indicates the name of the gene at which the probe is located. UCSC refgene group indicates the genomic region at which the probe is located. **a)** Sites with decrease in methylation were mined from the 2827 probes identified to be associated with DE exposure patterns. Sites were ordered from largest ∆β to smallest ∆β. **b)** Sites with increase in methylation were mined from the 2827 probes identified to be associated with DE exposure patterns. Sites were ordered from largest ∆β to smallest ∆β. **c)** Sites were mined from the 1118 probes with ≥15bp overlap with LINE1 elements in the genome. Sites were order from largest decrease in methylation to largest increase in methylation. **d)** Sites were mined from the 1271 probes with ≥15bp overlap with Alu elements in the genome. Sites were order from largest decrease in methylation to largest increase in methylation. Additional file 4: Figure S3. Changes in methylation of the *GSTP1* probe cg09038676 in response to DE-exposure stratified between subjects with the A allele and those with the G allele. Additional file 5: Figure S4. Mean beta value of exposure to FA at 0hr, 6hr, and 30hr for the 145 probes found to be significant for DE exposure but not for FA exposure. The beta value differences were first stratified between probes with decreased and increased methylation in response to DE, and then further divided between subjects who were exposed to DE first and those who were exposed to FA first.
