# Supplementary figures and images for: Short-term diesel exhaust inhalation in a controlled human crossover study is associated with changes in DNA methylation of circulating mononuclear cells in asthmatics
Source: Part Fibre Toxicol. 2014 Dec 9;11:71. doi: 10.1186/s12989-014-0071-3 (PMC4268899; doi:10.1186/s12989-014-0071-3)

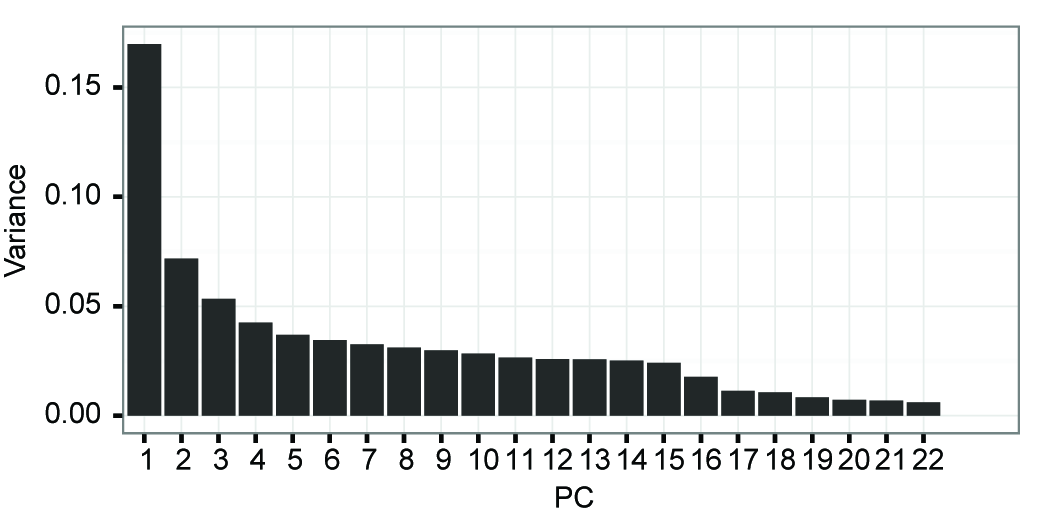

Supplement: Additional file 1: Figure S1. — Percent variance within the dataset accounted for by the first 22 principal components (0th principal component was disregarded since it is focused on probe offsets). [file 12989_2014_71_MOESM1_ESM.tiff]

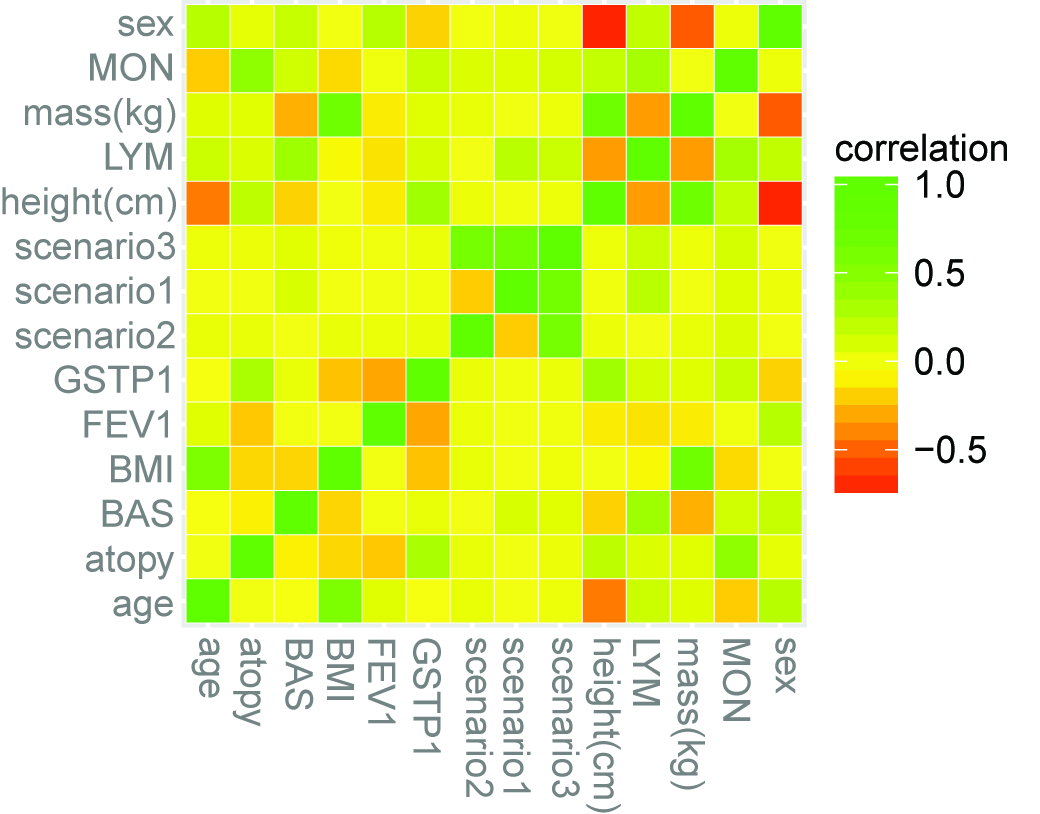

Supplement: Additional file 2: Figure S2. — Heatmap showing correlation among demographic variables and differential cell counts (please see Table 1 for units). Red indicates negative Pearson’s correlation coefficient of -0.5. Green indicates positive Pearson’s correlation coefficient of 1.0. Abbreviations are as follows: basophils (BAS), lymphocytes (LYM), monocytes (MON). [file 12989_2014_71_MOESM2_ESM.tiff]

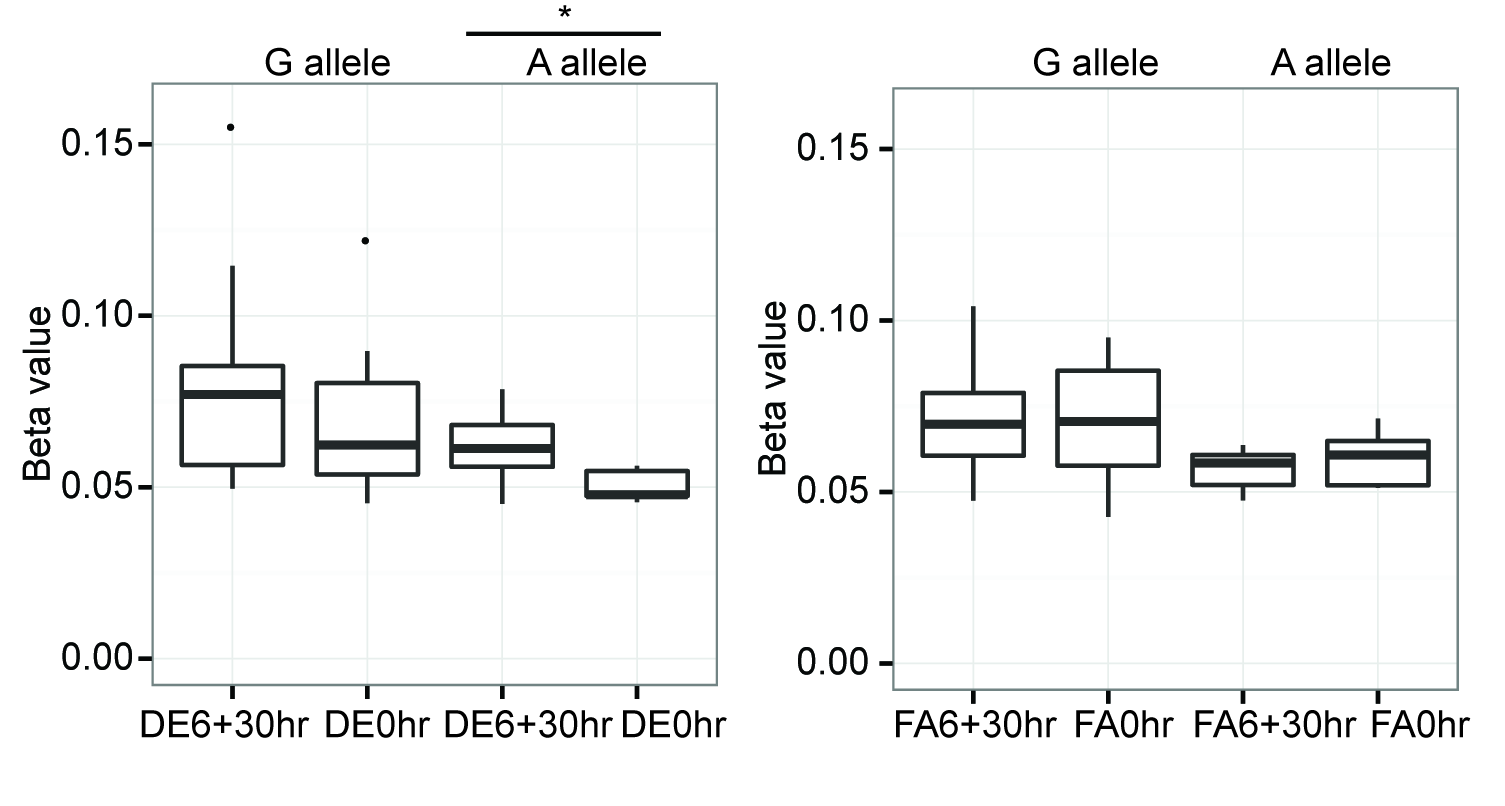

Supplement: Additional file 4: Figure S3. — Changes in methylation of the GSTP1 probe cg09038676 in response to DE-exposure stratified between subjects with the A allele and those with the G allele. [file 12989_2014_71_MOESM4_ESM.tiff]

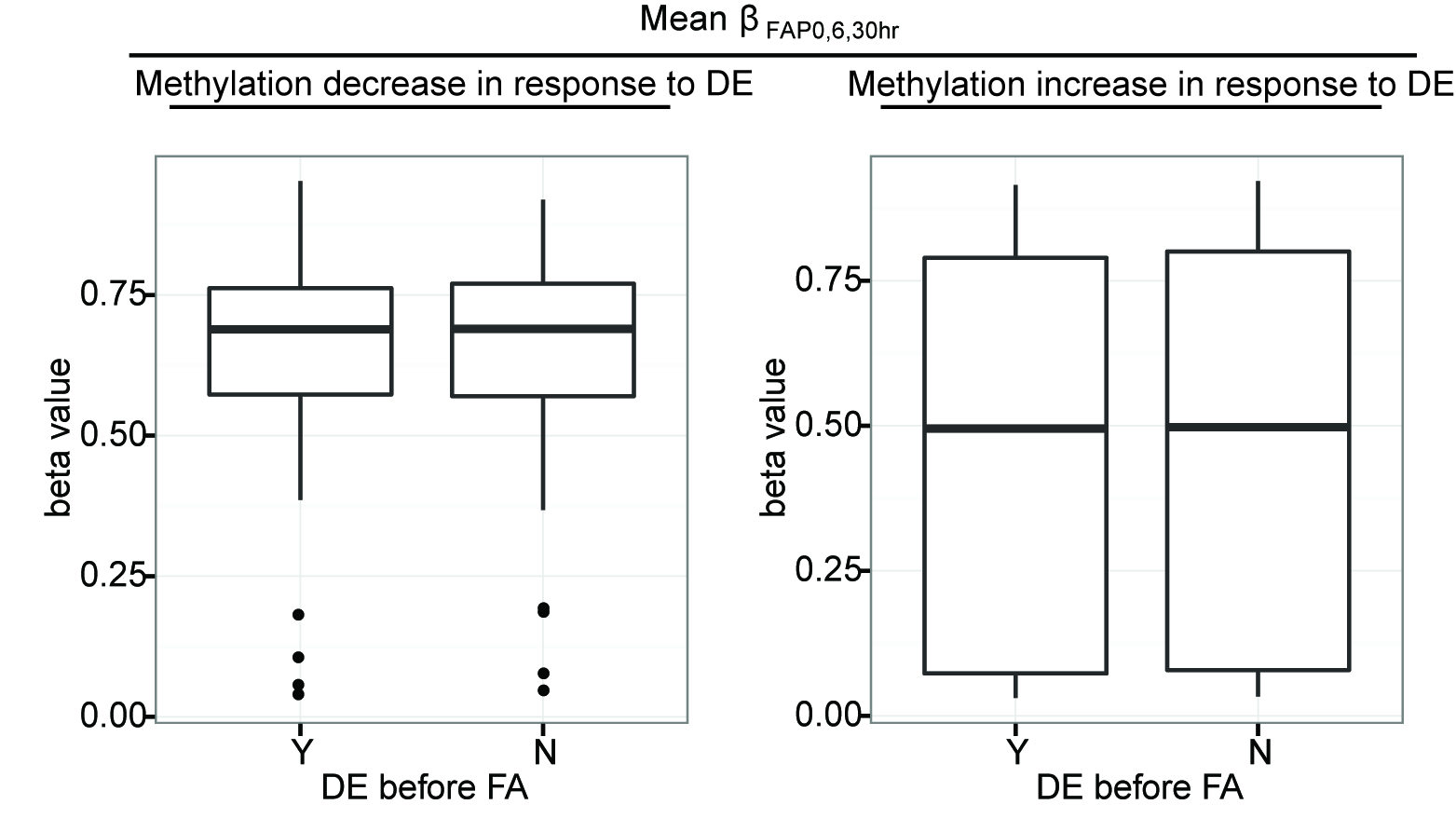

Supplement: Additional file 5: Figure S4. — Mean beta value of exposure to FA at 0hr, 6hr, and 30hr for the 145 probes found to be significant for DE exposure but not for FA exposure. The beta value differences were first stratified between probes with decreased and increased methylation in response to DE, and then further divided between subjects who were exposed to DE first and those who were exposed to FA first. [file 12989_2014_71_MOESM5_ESM.tiff]
