# Supplementary material for: Short-term diesel exhaust inhalation in a controlled human crossover study is associated with changes in DNA methylation of circulating mononuclear cells in asthmatics
Source: Part Fibre Toxicol. 2014 Dec 9;11:71. doi: 10.1186/s12989-014-0071-3 (PMC4268899; doi:10.1186/s12989-014-0071-3)
Supplement: Additional file 3: Table S1. — Top 4 DAVID functional annotation clusters. Enrichment of cellular component, biological process, and molecular function in the 2827 PCA filtered probes was calculated using all probes involved in this analysis as the background. Table S2. CpG sites found to have significant decrease in methylation as a result of DE exposure through LME modeling. For each probe, ∆β(FA6&30hr-FA0hr) represents the change in beta value from pre-FA exposure at 0hr and post-FA exposure at 6 and 30hr. ∆β(DE6&30hr-DE0hr) represents the change in beta value from pre-DE exposure to post-DE exposure. Closest TSS gene name indicates the gene name of the closest transcription start site. UCSC RefGene name indicates the name of the gene at which the probe is located. UCSC refgene group indicates the genomic region at which the probe is located. a) Sites with decrease in methylation were mined from the 2827 probes identified to be associated with DE exposure patterns. Sites were ordered from largest ∆β to smallest ∆β. b) Sites with increase in methylation were mined from the 2827 probes identified to be associated with DE exposure patterns. Sites were ordered from largest ∆β to smallest ∆β. c) Sites were mined from the 1118 probes with ≥15bp overlap with LINE1 elements in the genome. Sites were order from largest decrease in methylation to largest increase in methylation. d) Sites were mined from the 1271 probes with ≥15bp overlap with Alu elements in the genome. Sites were order from largest decrease in methylation to largest increase in methylation. [file 12989_2014_71_MOESM3_ESM.doc]

| a) | | | | | |
| --- | --- | --- | --- | --- | --- |
| **Illumina probe ID** | **Δβ(FA6&30hr-FA0hr)** | **Δβ(DE6&30hr-DE0hr)** | **Closest TSS gene name** | **UCSC RefGene name** | **UCSC refgene group** |
| cg27183818 | -0.038 | -0.069 | BCL10 |  |  |
| cg03917666 | -0.025 | -0.058 | AK055803 |  |  |
| cg20495370 | -0.023 | -0.058 | SLC7A7 | SLC7A7;SLC7A7;SLC7A7 | Body;Body;Body |
| cg14412134 | -0.037 | -0.057 | AKAP5 | MTHFD1 | Body |
| cg24414363 | -0.019 | -0.055 | CENPM | CENPM;CENPM;CENPM | TSS200;Body;Body |
| cg04211179 | -0.032 | -0.054 | ZBTB17 | ZBTB17 | 5'UTR |
| cg16266809 | -0.010 | -0.049 | ASXL1 |  |  |
| cg21664636 | -0.033 | -0.048 | BC015590 |  |  |
| cg27141509 | -0.011 | -0.047 | TRNA_Val |  |  |
| cg01965380 | -0.013 | -0.046 | LRRC8D | LRRC8D;LRRC8D | 5'UTR;5'UTR |
| cg16462648 | -0.016 | -0.044 | ANKRD55 |  |  |
| cg02156723 | -0.015 | -0.043 | MIIP |  |  |
| cg07462448 | -0.030 | -0.043 | CASP7 | CASP7;CASP7;CASP7;CASP7 | 5'UTR;5'UTR;5'UTR;5'UTR |
| cg23295629 | -0.027 | -0.042 | PVT1 | PVT1 | Body |
| cg26992245 | -0.013 | -0.042 | MIR3148 |  |  |
| cg19149314 | -0.003 | -0.042 | STK17B | HECW2 | 3'UTR |
| cg23304023 | -0.021 | -0.042 | TACC2 | TACC2;TACC2;TACC2;TACC2 | Body;Body;Body;Body |
| cg15006828 | -0.022 | -0.040 | BC100777 |  |  |
| cg11876705 | -0.015 | -0.040 | RASGRP4 | RASGRP4;RASGRP4;RASGRP4;RASGRP4;RASGRP4;RASGRP4;RASGRP4 | TSS1500;TSS1500;TSS1500;TSS1500;TSS1500;TSS1500;TSS1500 |
| cg03613942 | -0.019 | -0.039 | BC034940 |  |  |
| cg10063575 | -0.021 | -0.039 | AMHR2 | AMHR2;AMHR2;AMHR2 | Body;Body;Body |
| cg08131547 | -0.024 | -0.039 | ZNF121 | ZNF121 | 5'UTR |
| cg01464186 | -0.029 | -0.039 | SGMS1 | SGMS1 | 5'UTR |
| cg01894985 | 0.002 | -0.039 | MYLK | MYLK;MYLK;MYLK;MYLK | 5'UTR;5'UTR;5'UTR;5'UTR |
| cg08243465 | -0.015 | -0.039 | PMEPA1 | PMEPA1;PMEPA1;PMEPA1;PMEPA1 | 5'UTR;5'UTR;Body;Body |
| cg24458314 | -0.021 | -0.038 | HDAC9 | HDAC9;HDAC9;HDAC9;HDAC9;HDAC9 | Body;Body;Body;Body;Body |
| cg18986967 | -0.009 | -0.038 | AGK |  |  |
| cg07000713 | -0.027 | -0.037 | FAM19A2 | FAM19A2 | 5'UTR |
| cg19904265 | -0.021 | -0.036 | MIR4251 | PRDM16;PRDM16 | Body;Body |
| cg03655684 | 0.002 | -0.036 | CLCN7 | CLCN7;CLCN7 | Body;Body |
| cg01117384 | -0.010 | -0.036 | PMEPA1 | PMEPA1;PMEPA1;PMEPA1;PMEPA1 | 5'UTR;5'UTR;Body;Body |
| cg17212019 | -0.025 | -0.036 | NEFL |  |  |
| cg27457191 | -0.009 | -0.036 | PHTF2 | PHTF2;PHTF2 | 5'UTR;5'UTR |
| cg11465442 | -0.017 | -0.036 | DEFB136 | DEFB136 | TSS200 |
| cg11798406 | -0.017 | -0.036 | LINC00114 | NCRNA00114 | Body |
| cg08786003 | -0.015 | -0.035 | FCRL3 | FCRL3 | TSS200 |
| cg21585138 | -0.017 | -0.035 | CISH | CISH;CISH | Body;Body |
| cg10246903 | -0.021 | -0.035 | TSC22D1 |  |  |
| cg03999067 | -0.009 | -0.035 | U6 |  |  |
| cg23867673 | -0.011 | -0.035 | CDH23 | CDH23;CDH23 | Body;Body |
| cg03081173 | -0.021 | -0.035 | HCG27 | HCG27 | Body |
| cg25596287 | -0.017 | -0.034 | RCC1 | SNHG3-RCC1;RCC1;SNHG3-RCC1;RCC1;SNHG3-RCC1;RCC1 | Body;TSS1500;Body;TSS1500;5'UTR;TSS1500 |
| cg22930549 | 0.009 | -0.034 | Metazoa_SRP | RAD51L1;RAD51L1;RAD51L1 | Body;Body;Body |
| cg01613294 | -0.004 | -0.034 | APOL3 | APOL3;APOL3;APOL3;APOL3;APOL3;APOL3 | 5'UTR;TSS1500;TSS1500;TSS1500;TSS1500;5'UTR |
| cg26216876 | -0.012 | -0.033 | SLMO2 | SLMO2 | Body |
| cg02857074 | -0.008 | -0.033 | AK055932 | CACNA2D1 | Body |
| cg05462446 | -0.012 | -0.033 | ADAMTS18 |  |  |
| cg13461554 | -0.025 | -0.033 | LHCGR |  |  |
| cg13212186 | -0.023 | -0.032 | HCG27 | HCG27 | Body |
| cg25503381 | -0.020 | -0.032 | THBS1 | THBS1 | TSS1500 |
| cg11321190 | -0.024 | -0.032 | LRRFIP2 |  |  |
| cg19291696 | -0.022 | -0.032 | USP12 |  |  |
| cg21097090 | -0.008 | -0.031 | TNFAIP8 | TNFAIP8;TNFAIP8 | Body;Body |
| cg06190732 | 0.003 | -0.030 | SERPINA3 | SERPINA3 | TSS200 |
| cg07206827 | -0.009 | -0.030 | LINGO4 | LINGO4 | TSS1500 |
| cg17851868 | -0.019 | -0.030 | FLJ30838 |  |  |
| cg25812095 | -0.013 | -0.030 | GTDC1 | GTDC1;GTDC1 | 5'UTR;5'UTR |
| cg19222784 | -0.011 | -0.029 | NAV2 | NAV2;NAV2;NAV2;NAV2 | TSS1500;Body;Body;Body |
| cg05094429 | -0.006 | -0.029 | CCR6 | CCR6;CCR6 | 5'UTR;TSS200 |
| cg00465739 | -0.017 | -0.029 | ATP8A2 | ATP8A2 | Body |
| cg15464148 | -0.020 | -0.029 | LPAR5 | LPAR5 | 5'UTR |
| cg20427318 | -0.012 | -0.029 | LOC154092 |  |  |
| cg15192146 | -0.010 | -0.028 | AK098570 |  |  |
| cg08025405 | -0.008 | -0.028 | ASB3 |  |  |
| cg15718287 | -0.018 | -0.028 | DCLK1 | DCLK1 | 5'UTR |
| cg21536074 | -0.015 | -0.027 | GLI3 | GLI3 | Body |
| cg27305009 | -0.017 | -0.026 | WIPF1 | WIPF1;WIPF1 | 5'UTR;5'UTR |
| cg01890712 | -0.012 | -0.026 | OR9Q1 | OR9Q1 | TSS1500 |
| cg05033369 | -0.009 | -0.025 | FCRLA | FCRLA | TSS1500 |
| cg00135497 | -0.014 | -0.025 | LRAT |  |  |
| cg25298754 | -0.002 | -0.024 | ZBED2 | ZBED2;CD96;CD96 | 5'UTR;Body;Body |
| cg07376029 | -0.023 | -0.024 | GC | GC | TSS1500 |
| cg04230397 | -0.005 | -0.024 | MUC21 | MUC21 | Body |
| cg12159992 | -0.010 | -0.023 | MYBPC1 | MYBPC1;MYBPC1;MYBPC1;MYBPC1 | Body;Body;Body;Body |
| cg02989940 | -0.007 | -0.023 | AHSP | AHSP;AHSP | 5'UTR;1stExon |
| cg13262467 | -0.009 | -0.022 | LRRIQ4 | LRRIQ4 | TSS1500 |
| cg08241528 | -0.013 | -0.022 | RBPJ |  |  |
| cg25617519 | -0.013 | -0.022 | KLHL29 | KLHL29 | 5'UTR |
| cg16455376 | -0.007 | -0.021 | CARHSP1 |  |  |
| cg05246530 | -0.003 | -0.021 | ST3GAL2 | ST3GAL2 | 5'UTR |
| cg02046532 | -0.008 | -0.021 | DEFB129 | DEFB129 | TSS200 |
| cg25609393 | -0.003 | -0.021 | HCN1 | HCN1 | Body |
| cg23670794 | 0.000 | -0.020 | ZBED2 | ZBED2;CD96;CD96 | 5'UTR;Body;Body |
| cg26207423 | 0.003 | -0.020 | HMBOX1 | HMBOX1;HMBOX1 | 5'UTR;5'UTR |
| cg01919768 | -0.007 | -0.020 | TFR2 | TFR2 | TSS1500 |
| cg15150463 | -0.010 | -0.020 | GJA10 |  |  |
| cg17365725 | -0.014 | -0.020 | D2HGDH | D2HGDH | Body |
| cg06106484 | -0.004 | -0.019 | TRNA_Pseudo | |  |
| cg18447402 | -0.009 | -0.019 | MRPS33 | MRPS33;MRPS33 | Body;Body |
| cg07157117 | -0.006 | -0.019 | AGXT2L1 |  |  |
| cg13583523 | -0.008 | -0.018 | LOC340107 |  |  |
| cg26556196 | 0.001 | -0.018 | KHDRBS2 | KHDRBS2 | Body |
| cg18837292 | -0.005 | -0.018 | IRX1 |  |  |
| cg27495728 | -0.003 | -0.016 | TRNA_Trp |  |  |
| cg21071237 | 0.005 | -0.014 | ADAMTS15 |  |  |
| cg02634628 | -0.004 | -0.013 | UNCX |  |  |
| cg23652785 | -0.010 | -0.011 | AF086258 |  |  |
| cg07229076 | -0.003 | -0.008 | ARL4A | ARL4A;ARL4A;ARL4A | 5'UTR;TSS200;5'UTR |
| cg04135246 | 0.001 | -0.008 | BC038465 |  |  |
| cg04204002 | 0.002 | -0.007 | TMPRSS3 | TMPRSS3;TMPRSS3 | TSS1500;TSS1500 |
| cg07221635 | 0.000 | -0.005 | PECR | TMEM169;PECR;TMEM169;TMEM169;TMEM169 | TSS200;1stExon;TSS200;TSS200;TSS200 |
| cg11351779 | 0.003 | -0.003 | MPPED1 | MPPED1 | 5'UTR |

| **b)** | | | | | |
| --- | --- | --- | --- | --- | --- |
| **Illumina probe ID** | **Δβ(FA6&30hr-FA0hr)** | **Δβ(DE6&30hr-DE0hr)** | **Closest TSS gene name** | **UCSC refgene name** | **UCSC refgene group** |
| cg18944752 | 0.020 | 0.057 | RGS12 | RGS12;RGS12;RGS12 | Body;Body;Body |
| cg22331200 | 0.007 | 0.045 | MPO | MPO | Body |
| cg10049789 | 0.020 | 0.043 | SH3TC1 | SH3TC1 | 5'UTR |
| cg23756272 | 0.024 | 0.039 | BCL2 | BCL2 | Body |
| cg04309234 | 0.014 | 0.036 | PRDM1 |  |  |
| cg09729012 | 0.016 | 0.036 | RYBP |  |  |
| cg07142009 | 0.016 | 0.033 | XAF1 |  |  |
| cg04109092 | 0.014 | 0.033 | TTYH3 | IQCE;IQCE | Body;Body |
| cg25437886 | 0.010 | 0.033 | LIMD1 | LIMD1 | Body |
| cg26060971 | 0.012 | 0.032 | DNAH1 | DNAH1 | Body |
| cg25150953 | 0.010 | 0.032 | LIMCH1 | LIMCH1;LIMCH1;LIMCH1 | Body;Body;Body |
| cg09597638 | 0.006 | 0.032 | AB062083 |  |  |
| cg06478504 | 0.017 | 0.031 | HDAC4 | HDAC4 | Body |
| cg13029400 | 0.005 | 0.029 | ZBTB38 | ZBTB38 | 5'UTR |
| cg14440934 | 0.026 | 0.029 | ZDHHC1 | ZDHHC1 | TSS1500 |
| cg17624536 | 0.015 | 0.025 | PRDM1 |  |  |
| cg01201279 | 0.012 | 0.024 | AK094480 | LEKR1 | TSS1500 |
| cg25158320 | 0.003 | 0.024 | SERPINF1 | SERPINF1;SERPINF1 | 1stExon;5'UTR |
| cg11301250 | 0.001 | 0.023 | VEPH1 | VEPH1;VEPH1;VEPH1;VEPH1;VEPH1 | TSS200;5'UTR;5'UTR;5'UTR;5'UTR |
| cg05084827 | 0.013 | 0.023 | RPS27A | C2orf63;C2orf63 | Body;Body |
| cg16887334 | 0.005 | 0.021 | OXT | OXT | TSS200 |
| cg15600238 | 0.000 | 0.021 | HOTTIP |  |  |
| cg06561886 | 0.009 | 0.020 | SLC44A2 | SLC44A2;SLC44A2;SLC44A2 | 5'UTR;1stExon;Body |
| cg27380292 | 0.004 | 0.018 | MIR4499 |  |  |
| cg19931644 | 0.005 | 0.018 | LONRF1 |  |  |
| cg09322534 | 0.011 | 0.015 | SLC2A1 |  |  |
| cg09841842 | 0.005 | 0.015 | U6 | FRMD6 | 5'UTR |
| cg12502403 | 0.002 | 0.014 | MARK2 | MARK2;MARK2;MARK2;MARK2 | 1stExon;1stExon;1stExon;1stExon |
| cg07673080 | -0.007 | 0.014 | MIR4269 | HDAC4 | Body |
| cg26018827 | 0.004 | 0.013 | GEN1 | GEN1;SMC6;GEN1;SMC6;GEN1 | 5'UTR;TSS1500;1stExon;TSS1500;5'UTR |
| cg24291500 | 0.000 | 0.013 | REST | REST | TSS1500 |
| cg08260406 | -0.008 | 0.012 | OR2L13 | OR2L13 | TSS200 |
| cg09038676 | -0.001 | 0.010 | GSTP1 | GSTP1 | Body |
| cg16716750 | 0.000 | 0.010 | RGS17 | RGS17 | 5'UTR |
| cg20291033 | 0.004 | 0.010 | TNR | TNR | TSS200 |
| cg20102877 | -0.002 | 0.009 | KRTCAP3 | KRTCAP3;KRTCAP3 | Body;Body |
| cg17154022 | 0.003 | 0.007 | HMGN2 | HMGN2;HMGN2 | 1stExon;5'UTR |
| cg11960393 | 0.003 | 0.007 | LOX | LOX | TSS200 |
| cg26802256 | 0.003 | 0.006 | FOXK1 |  |  |
| ch.13.32987270F | 0.001 | 0.006 | STARD13 |  |  |
| ch.7.107350695F | -0.001 | 0.004 | DLD |  |  |
| cg11798043 | -0.002 | 0.004 | LMBRD1 | LMBRD1 | Body |
| cg26056770 | 0.001 | 0.003 | TMEM141 | TMEM141 | TSS1500 |

| **c)** | | | | | |
| --- | --- | --- | --- | --- | --- |
| **Illumina probe ID** | **Δβ(FA6&30hr-FA0hr)** | **Δβ(DE6&30hr-DE0hr)** | **Closest TSS gene name** | **UCSC refgene name** | **UCSC refgene group** |
| cg11075561 | -0.031 | -0.043 | MAPK13 |  |  |
| cg05266321 | -0.007 | -0.028 | CCR2 | CCR2 | 3'UTR |
| cg17729072 | -0.012 | -0.026 | AK092451 |  |  |
| cg04426653 | -0.003 | -0.025 | BC033989 |  |  |
| cg15210817 | -0.016 | -0.024 | CSF3R | CSF3R;CSF3R;CSF3R;CSF3R | TSS1500;TSS1500;TSS1500;TSS1500 |
| cg02224002 | -0.008 | -0.024 | RNF166 | RNF166 | Body |
| cg10136452 | -0.011 | -0.023 | BC087858 |  |  |
| cg25840780 | -0.009 | -0.022 | NFATC2 | NFATC2;NFATC2;NFATC2 | Body;Body;Body |
| cg22689597 | -0.006 | -0.022 | LRRC10 | LRRC10 | TSS1500 |
| cg14248598 | -0.011 | -0.021 | C1S |  |  |
| cg25619459 | -0.016 | -0.020 | CDK14 | CDK14 | Body |
| cg05392244 | -0.007 | -0.019 | FOXC1 |  |  |
| cg21874404 | -0.003 | -0.017 | SNORD112 | MEG8 | Body |
| cg20728419 | -0.006 | -0.013 | USP47 | USP47 | Body |
| cg21750986 | -0.006 | -0.013 | RAB12 |  |  |
| cg12154976 | -0.005 | -0.012 | RASGEF1C | RASGEF1C | 5'UTR |
| cg01830883 | -0.005 | -0.012 | WRAP73 | WDR8 | Body |
| cg02713720 | -0.001 | -0.010 | BC152379 | FBLN2;FBLN2;FBLN2 | Body;Body;Body |
| cg19587838 | -0.002 | 0.009 | SOX1 |  |  |
| cg03993154 | 0.005 | 0.012 | SLC13A1 | SLC13A1 | Body |
| cg09904296 | 0.001 | 0.019 | GOLGA3 | GOLGA3 | TSS1500 |
| cg20860806 | 0.004 | 0.019 | SHC4 |  |  |
| cg09500196 | 0.010 | 0.020 | CARHSP1 | CARHSP1 | TSS1500 |
| cg02215171 | 0.007 | 0.028 | HERC5 | HERC5 | Body |
| cg17554875 | 0.014 | 0.028 | RNF130 |  |  |
| cg12930304 | 0.015 | 0.028 | SERPINB1 |  |  |
| cg27220070 | -0.001 | 0.031 | EP400 | EP400 | TSS1500 |
| cg12930392 | 0.005 | 0.032 | PAK2 | PAK2 | 5'UTR |
| cg10225149 | 0.010 | 0.034 | AKAP8 | AKAP8L;AKAP8 | Body;TSS1500 |
| cg18437319 | 0.006 | 0.034 | MCAT |  |  |
| cg13428066 | 0.013 | 0.044 | KCNQ1OT1 | KCNQ1;KCNQ1OT1;KCNQ1 | Body;Body;Body |

| **d)** | | | | | |
| --- | --- | --- | --- | --- | --- |
| **Illumina probe ID** | **Δβ(FA6&30hr-FA0hr)** | **Δβ(DE6&30hr-DE0hr)** | **Closest TSS gene name** | **UCSC refgene name** | **UCSC refgene group** |
| cg07128021 | -0.018 | -0.039 | LOC283050 | LOC283050;LOC283050;LOC283050 | Body;Body;Body |
| cg21593149 | -0.017 | -0.038 | MAEA | MAEA;MAEA | Body;Body |
| cg08343600 | -0.015 | -0.033 | HNRNPF | HNRNPF;HNRNPF;HNRNPF;HNRNPF;HNRNPF | 5'UTR;TSS1500;5'UTR;5'UTR;5'UTR |
| cg13502540 | -0.021 | -0.031 | HCG27 | HCG27 | Body |
| cg22553301 | -0.004 | -0.030 | ZFR2 | ZFR2 | Body |
| cg20130098 | -0.017 | -0.025 | KALRN | KALRN;KALRN | Body;Body |
| cg26785702 | -0.020 | -0.025 | FOXR1 | FOXR1 | Body |
| cg06482498 | -0.010 | -0.023 | C6orf48 | C6orf48;SNORD48;C6orf48 | TSS1500;TSS1500;TSS1500 |
| cg05669244 | -0.009 | -0.022 | ZNF358 |  |  |
| cg16747928 | -0.009 | -0.021 | AK092451 |  |  |
| cg02188939 | -0.007 | -0.020 | SLC43A2 | SLC43A2 | Body |
| cg08061463 | -0.010 | -0.020 | TTLL13 |  |  |
| cg02403031 | -0.007 | -0.016 | Mir_633 |  |  |
| cg22635673 | 0.006 | 0.010 | BHLHA9 |  |  |
| cg03148858 | 0.006 | 0.014 | TLE2 | TLE2 | TSS1500 |
| cg23482747 | 0.004 | 0.015 | ARRDC2 | ARRDC2 | Body |
| cg01801040 | -0.001 | 0.016 | TTC19 | ZSWIM7;ZSWIM7 | Body;Body |
| cg00030420 | 0.011 | 0.019 | SLC25A2 | SLC25A2 | TSS200 |
| cg08812692 | 0.007 | 0.021 | CLK4 | CLK4 | 5'UTR |
| cg00408567 | 0.003 | 0.028 | FLJ38109 | GALNT10;GALNT10 | Body;Body |
| cg13443575 | 0.009 | 0.033 | SLFN13 | SLFN13 | TSS200 |
| cg17653886 | 0.013 | 0.035 | SLFN13 | SLFN13 | TSS1500 |
| cg04354393 | 0.016 | 0.035 | SLFN13 | SLFN13 | TSS200 |
| cg00943851 | 0.015 | 0.036 | AK092451 |  |  |
| cg08866695 | 0.017 | 0.041 | ACACB | ACACB | Body |
